# Potential Microbiological Risk Factors Associated With Periodontitis and Periodontal Health Disparities

**DOI:** 10.3389/fcimb.2021.789919

**Published:** 2021-11-17

**Authors:** Bing-Yan Wang, Tom Lu, Qiuyin Cai, Meng-Hsuan Ho, Sally Sheng, Hsiu-Wan Meng, Laura Arsto, Jianming Hong, Hua Xie

**Affiliations:** ^1^ School of Dentistry, University of Texas Health Science Center at Houston, Houston, TX, United States; ^2^ Department of Mathematics and Statistics, Texas Tech University, Lubbock, TX, United States; ^3^ Division of Epidemiology, Department of Medicine, Vanderbilt Epidemiology Center, Vanderbilt-Ingram Cancer Center, Vanderbilt University School of Medicine, Nashville, TN, United States; ^4^ School of Dentistry, Meharry Medical College, Nashville, TN, United States

**Keywords:** *Porphyromonas gingivalis*, periodontitis, racial and ethnic groups, *Streptococcus cristatus*, microbial profiles

## Abstract

Periodontitis disproportionately affects different racial and ethnic populations. In this study, we used qPCR to determine and compare oral microbial profiles in dental plaque samples from 191 periodontitis patients of different ethnic/racial backgrounds. We also obtained the periodontal parameters of these patients retrospectively using axiUm and performed statistical analysis using SAS 9.4. We found that in this patient cohort, neighborhood median incomes were significantly higher among Caucasians Americans (CAs) than among African Americans (AAs) and Hispanic Americans (HAs). Levels of total bacteria and *Porphyromonas gingivalis*, a keystone periodontal pathogen, were not evenly distributed among the three groups. We confirmed our previous findings that *Streptococcus cristatus* reduces *P. gingivalis* virulence potential and likely serves as a beneficial bacterium. We also showed the ratio of *S. cristatus* to *P. gingivalis* to be significantly higher in CAs than in HAs and AAs. Our results suggest that higher levels of *P. gingivalis* and lower ratios of *S. cristatus* to *P. gingivalis* may contribute to periodontal health disparities.

## Introduction

Periodontitis is recently defined as a dysbiotic disease resulting from imbalanced oral microbiota (Hajishengallis and Lamont, 2021). The etiology of periodontitis has been developed from microbial accumulation and specific periodontal pathogens to keystone pathogen-associated polymicrobial dysbiosis (Socransky and Haffajee, 1994; Hajishengallis et al., 2012; Hajishengallis and Lamont, 2012). *Porphyromonas gingivalis*, a gram-negative bacterium, plays a vital role in the development of dysbiotic microbial communities. While a low-abundance species in oral microbial communities, *P. gingivalis* can act in concert with other microbes to disrupt host-microbial homeostasis and induce uncontrolled inflammatory responses in periodontal tissues (Hajishengallis et al., 2011; Hajishengallis and Lamont, 2021).

The National Health and Nutrition Examination Survey (NHANES) 2009–2014 revealed the prevalence of periodontitis among dentate adults aged 30 years and older to be significantly different among African Americans (AAs), Caucasian Americans (CAs), and Hispanic Americans (HAs) (Eke et al., 2018). AAs and HAs exhibit much a higher incidence of periodontitis than CAs, a phenomenon evident even in populations with severe periodontitis. We observed higher detection rates of *P. gingivalis* in AAs and HAs than in CAs, diagnosed either as periodontal health or with biofilm-induced gingivitis on an intact periodontium (unpublished data). We also found that most *P. gingivalis* detected in AAs were of *fimA* genotypes II and IV that are associated with higher plaque indexes and levels of bleeding on probing in an intact periodontium cohort (unpublished data). Based on these observations, we hypothesized that differential oral microbial profiles exist in periodontitis patients of different racial/ethnic backgrounds. Here, we investigated a potential link between microbial composition, particularly the prevalence of *P. gingivalis* and *Streptococcus cristatus*, and periodontitis risks among AAs, CAs, and HAs. Our results suggest that differences in the microbial composition of dental biofilms may influence the initiation of periodontitis, and that individuals susceptible to periodontitis may depend, to some extent, on the microbial composition of early dental biofilm colonizers such as *S. cristatus*.

## Materials and Methods

### Study Cohorts

The research protocol was approved by the Committee for the Protection of Human Subjects of the University of Texas Health Science Center at Houston (UTHealth at Houston) (IRB number: HSC-DB-17-0636). Candidates were screened during routine dental visits at the clinic of School of Dentistry at UTHealth at Houston between 2018 and 2021. Individuals aged 21–75 with self-reported ethnicity/race of non-Hispanic African American (AA), non-Hispanic Caucasian American (CA), or Hispanic American (HA) were enrolled after initial periodontal examination. The examination documented plaque index (PI), bleeding on probing (BOP), probing depth (PD), clinical attachment level (CAL), furcation involvement, tooth mobility, and keratinized tissue on all teeth (Newman et al., 2018). Radiographs were taken during this screening phase to assess bone loss. The clinical periodontal examinations were performed by trained dental examiners who are faculty members of the School of Dentistry, UTHealth at Houston. All study participants were diagnosed with generalized periodontitis Stage II or III, regardless of their grading, based on the 2017 World Workshop classification (Tonetti et al., 2018; Papapanou et al., 2018a). The enrolled patients also met the following criteria: ≤4 tooth loss due to periodontitis, interdental CAL ≥3mm and PD ≥5 mm at two or more teeth in different quadrants, and radiographic bone loss ≥15%. Other criteria for study participation were 1) no scaling and root planning within the previous year or periodontal surgeries in the previous five years; 2) no antibiotic therapy in the previous six months; 3) not pregnant. The periodontal parameters and diagnosis of participants were abstracted from the Electronic Health Record (axiUm). In addition, medical history and dental history were extracted from axiUm and recorded by a dental student (LA) who is blinded to study design and bacterial data. Cardiovascular diseases (including infective endocarditis, coronary artery disease, heart attach, congestive heart failure, hypertension, cardiac arrhythmia, rheumatic fever, stroke, mitral valve prolapse), respiratory diseases (chronic bronchitis or emphysema, asthma), sleep apnea, renal failure, gastrointestinal diseases (including hepatitis, stomach ulcer, crohn’s disease, colitis, liver disease), endocrine diseases (diabetes, thyroid disorder), hemotologic disorders, arthritis, osteoporosis, depression, autoimmune diseases, cancer, usage of alcohol or tobacco, dry mouth, and habitually clench or grind teeth were recorded as present or abscent. Body mass index (BMI) was recorded as numerical numbers. Neiberhood median incomes of the participants were decided based on their residential zip codes (https://www.incomebyzipcode.com/).

### Plaque Sample Collection

Dental plaque samples were collected by board-certified periodontists using sterile paper points at baseline prior to any dental treatment. The samples were labelled with numbers according to sampling sequence. The paper points were placed in ≥5mm pockets in different quadrant for 1 minute and then immersed immediately in an Eppendorf tube with 0.5 ml of Tris-EDTA (TE) buffer (pH 7.5) (Wang et al., 2009). Oral bacteria were harvested by centrifugation and the bacterial pellets were resuspended in 100 µl TE buffer. Chromosomal DNA was released by two cycles of freezing at -80^°^C overnight and boiling for 10 minutes.

### Bacterial Quantitation by qPCR

All bacteria tested were enumerated by qPCR using SYBR Green PCR mix (Bio-Red Laboratories Inc., Redmond, WA, USA) with species-specific primers listed in **Table 1**. P*. gingivalis* strains with different *fimA* genotypes were identified by qPCR using strain-specific primers (Zheng et al., 2011). Levels of total bacteria were determined using primers corresponding to the conserved sequences of cyanobacterial small subunit rRNA genes (Turner et al., 1999) (**Table 1**). Standard curves used to enumerate bacterial cells were generated by qPCR using the genomic DNA from each bacterial species as previously described (Wang et al., 2009). Bacterial ratios were calculated using the numerical levels of two bacteria from the same sample. Bacterial quantitation by qPCR was performed by a designated technician blinded to subject information.

**Table 1:** Primers used in this study.

Gene	Primer sequences (5’-3’)
*Cyanobacterial 16S rRNA*	GGGCTACACACGYGCWAC GACGGGCGGTGTGTRCA
*S. cristatus arcA*	CTGACGAAGCGAAAGGTCTG ATGTGGTTGAGCGATACAGC
*S. gordonii 16S-rRNA*	CCACACTGGGACTGAGACAC TGCTCGGTCAGACTTTCGTC
*P. gingivalis16s-rRNA*	TGTAGATGACTGATGGTGAAA ACTGTTAGCAACTACCGATGT
*P. gingivalis fimA I*	CTGTGTGTTTATGGCAAACTTC AACCCCGCTCCCTGTATTCCGA
*P. gingivalis fimA Ib*	CTCTTAAGATCAAGCGTGTA TGTCAGATAATTAGCGTCTCG
*P. gingivalis fimA II*	AACCCCGCTCCCTGTATTCCGA ACAACTATACTTATGACAATGG
*P. gingivalis fimA III*	ATTACACCTACACAGGTGAGGC AACCCCGCTCCCTGTATTCCGA
*P. gingivalis fimA IV*	CTATTCAGGTGCTATTACCCAA AACCCCGCTCCCTGTATTCCGA
*P. gingivalis fimA V*	AACAACAGTCTCCTTGACAGTG TATTGGGGGTCGAACGTTACTGTC
*F. nucleatum 16S-rRNA*	ACGTATGTCACGAGCGTTATC CTTGTAGTTCCGCTTACCTCTC
*T. forsythia 16S-rRNA*	GAGGAAGGTCCCCCACACTG CTGGCACGGAGTTAGCCGAT
*T. denticola 16S-rRNA*	GGCGGTTAGGTAAGCCTGGT CCGGTTTCCCCTCCGTGATT

### Statistical Analysis

Continuous variables were analyzed using one-way ANOVA. The chi-squared test or Fisher’s exact test was performed for categorical variables. Levels of periodontitis-associated bacteria were categorized into two groups by their medians and compared among the three study groups. Stratified analyses were conducted to evaluate the differences in levels of periodontitis-associated bacteria between perodontitis stages II and III. Correlations between levels of bacterial species (*r*) were determined using Pearson correlation coefficient. P-values <0.05 were considered statistically significant. SAS version 9.4 (SAS Institute, Cary, NC, USA) was used to conduct all statistical analyses.

## Results

### Clinical Characteristics of the Study Cohort

We enrolled 191 periodontitis patients in this study, including 56 AAs, 67 CAs, and 68 HAs with the mean age of 51.66 ± 12.79 years. Diagnosis creteria for the patients were based on the 2017 World Workshop classification (Tonetti et al., 2018; Papapanou et al., 2018b). Among them, 61.78% were diagnosed with periodontitis stage III and 38.22% were diagnosed with stage II. There was no significant difference in the distribution of periodontitis stages among AAs (66.04% with stage III), CAs (54.41% with stage III), and HAs (67.64% with stage III) (*p* = 0.291). In addition, there was no significant difference among the racial and ethnic groups with regards to gender (*p* = 0.228). To investigate the possible existence of social inequalities among these racial/ethnic groups, we used the participants’ residential zip codes to obtain median incomes of the neighborhoods in which they reside. Our results showed the neighborhood median income of the CA group to be significantly higher ($73,895 ± $26,906) than that of the AA or HA group ($58,013 ± 22,470 and $58,558 ± $ 21,434, respectively) (*p*<0.001). Our observation agrees with a previously reported likelihood of an association between neighborhood socioeconomic circumstances and periodontitis (Borrell et al., 2006). However, further studies are needed to elucidate how economic conditions of a neighborhood impact periodontal health of its residents.

We also found significant differences in levels of BOP among the racial/ethnic groups. We observed the highest degree of BOP in the HA group (44.18% ± 25.80%), followed by the AA group (41.91% ± 25.74%) and the CA group (38.39% ± 24.51%) (*p* = 0.007) (**Table 2**). However, we did not observe significant differences in the levels of PI among AAs (66.61% ± 32.27%), CAs (61.65% ± 24.57%), and HAs (64.77% ± 27.57%) (*p* = 0.569). These results suggest that different profiles of microbiota likely led to a more severe inflammatory response found in AA and HA patients compared to CA patients.

**Table 2:** Periodontal characteristics of the study cohort.

Racial/ethnic groups	AA	CA	HA	Total	*P* – value
Periodontal evaluation					
BOP (%)^a^	41.91 ± 25.74	38.39 ± 24.51	51.75 ± 25.61	44.18 ± 25.80	0.007
PI (%)^b^	66.61 ± 32.27	61.65 ± 24.57	66.46 ± 26.70	64.77 ± 27.57	0.569
Periodontitis stages^c^					
II	18	31	22	71	
III	35	37	46	118	0.291
Tooth number (Mean ± SD)^d^	25.89 ± 4.08	25.63 ± 3.51	27.19 ± 2.55	26.26 ± 3.45	0.019

^a^BOP, Bleeding on Probing; ^b^PI, Modified O’Leary plaque Index; ^c^Periodontitis stages: Based on 2017 World Workshop classification; ^d^Based on 32 teeth.

### Microbial Profiles of Dental Plaques From the Study Cohort

To investigate the oral microbial profiles of the study subjects, we used qPCR to measure the distribution and levels of several well-studied oral bacteria in their dental plaque samples, including keystone pathogens, accessory pathogens, and pathobionts (Hajishengallis and Lamont, 2016). Since the data are not evenly distributed, we designated bacterial levels as either higher or lower than their medians. As shown in **Table 3**, total bacterial levels in the dental plaque samples were determined using primers corresponding to cyanobacterial 16S rRNA as probes (Turner et al., 1999). Approximately 50% of all samples had more than 10^9^ total bacterial cells. However, 71% of samples from the AA patients had more than 10^9^ total bacterial cells, which was much higher than that for the CA patients (47%) and for the HA patients (38%) (*p* = 0.0009). Using qPCR, we detected *P. gingivalis* in all 191 samples. Higher levels of *P. gingivalis* (>10^6^) was detected in 54.41% of samples from the HA patients and in 48.21% of samples from the AA patients, but in only 24.88% of samples from the CA patients (*p* = 0.0008), which may be linked to higher BOP index observed in the HA and AA patients. We also detected *Tannerella forsythia* and *Fusobacterium nucleatum* in all samples, while *Treponema denticola* was present in 66.49% of the samples. These three species were all evenly distributed among the AA, CA, and HA groups.

**Table 3:** Distributions of periodontitis-associated bacteria in samples from patients of different racial/ethnic backgrounds.

Bacterial level	Bacterial prevalence (%)				
	All	AA	CA	HA	*P*-value
Total bacteria	100				
<10^9^	48.69	28.57	52.24	61.76	0.0009
>10^9^	51.30	71.43	47.76	38.24	
*P. gingivalis* (*Pg*)	100	100	100	100	
<10^6^	58.1	51.79	76.12	45.59	0.0008
>10^6^	41.9	48.21	23.88	54.41	
*T. forsythia* (*Tf*)	100	100	100	100	
<5x10^5^	45.55	39.29	50.75	45.59	0.445
>5x10^5^	54.45	60.71	49.25	54.41
*T. denticola* (*Td*)	66.49	71.43	59.70	69.12	
<10^3^	40.9	42.50	45.00	36.17	0.685
>10^3^	59.1	57.50	55.00	63.83	
*F. nucleatum*	100	100	100	100	
<2.5x10^6^	53.43	42.86	59.70	55.88	0.154
>2.5x10^6^	46.59	57.14	40.30	44.12	
*S. cristatus*/*Pg*	100	100	100	100	
<100	60.21	58.93	49.25	72.06	0.025
>100	39.79	41.07	50.75	27.94	
*S. cristatus*/*Tf*	100	100	100	100	
<100	82.20	80.36	86.57	79.41	0.505
>100	17.80	19.64	13.43	20.59	
*S. cristatus*/*Td*	100	100	100	100	
<1000	33.07	27.50	42.50	29.79	0.301
>1000	66.93	72.50	57.50	70.21	

Our previous study on 13 subjects with periodontitis demonstrated that there appeared to be an inverse relationship between the number of *S. cristatus* versus *P. gingivalis* cells in dental plaque (Wang et al., 2009). Therefore, we postulated that higher levels of *S. cristatus*, an early colonizer of oral microbial communities, may control and suppress *P. gingivalis* levels, and that the higher risk of periodontitis in AA and HA populations is, at least in part, dependent on a different microbial composition containing less *S. cristatus*. Here, we found that 50.75% of dental plaque samples from the CA patients had *S. cristatus*/*P. gingivalis* ratios greater than 100, compared to 41.07% of samples from the AA patients and 27.94% of those from the HA patients (*p* = 0.025) (**Table 3**). Further analysis of the *S. cristatus*/*P. gingivalis* ratios revealed a significant difference in the levels of *P. gingivalis* and *T. denticola* between dental plaque samples with higher *S. cristatus*/*P. gingivalis* ratios and samples with lower ratios (**Table 4**). Particularly, levels of *P. gingivalis* were approximately 700 time higher in samples with relatively lower levels of *S. cristatus*. We also observed a slight increase in the abundance of total bacteria, *T. denticola*, and *F. nucleatum* in samples with lower *S. cristatus/P. gingivalis* ratios. These results further support our postulation that *S. cristatus* benefits the host by antagonizing the colonization and accumulation of *P. gingivalis* (Wang et al., 2009).

**Table 4:** Impact of *T. denticola* and the *S. cristatus/P. gingivalis* ratios on the abundance of periodontitis-associated species.

Mean of bacteria						
Bacteria	With *T. denticola*	Without *T. denticola*	*P* - value	*S. cristatus/P. gingivalis*>100	*S. cristatus/P. gingivalis<*100	*P* - value
Total bacteria	5.04 × 10^9^	3.27 × 10^9^	0.201	3.48 × 10^9^	5.09 × 10^9^	0.2435
*P. gingivalis*	8.87 × 10^7^	5.10 × 10^6^	0.0078	1.39 × 10^5^	1.01 × 10^8^	0.0037
*T. forsythia*	2.33 × 10^6^	1.67 × 10^6^	0.1866	1.44 × 10^6^	2.55 × 10^6^	0.0209
*T. denticola*				2.49 × 10^4^	3.13 × 10^4^	0.7349
*F. nucleatum*	3.95 × 10^6^	4.13 × 10^6^	0.8284	3.84 × 10^6^	4.12 × 10^6^	0.6991

In addition, we examined the distribution of *P. gingivalis* of different *fimA* genotypes (types I and Ib–V), which are classified based on their nucleotide sequences of the *fimA* gene. We found *P. gingivalis* of *fimA* genotype II to be present in 55.5% of all samples. This is consistent with previous reports stating *P. gingivalis* type II as the predominant *P. gingivalis* strain found in periodontitis patients (Amano et al., 1999; Enersen et al., 2008). We also detected the type IV strain in 20.94% of all samples, followed by type III (10.47%), type I (9.94%), type Ib (2.10%), and type V (2.09%) (**Table 5**). We did not observe any significant difference in the distribution of the different *fimA* strains among the three racial/ethnic groups and between patients with periodontitis stage II and III.

**Table 5:** Distribution of *P. gingivalis fimA* types in periodontitis patients.

Variables	Occurrence of the *fimA* types (%)						
	I	Ib	II	III	IV	V	*P*-value
Groups							
All participants	9.94	2.10	55.50	10.47	20.94	2.09	
AA	12.50	1.79	57.1	7.14	19.6	1.78	0.939
CA	8.96	1.49	53.7	10.4	25.4	0	
HA	8.82	2.94	55.9	13.2	17.6	1.47	
Periodontitis Stage							
Stage II	12.68	0	54.93	9.86	19.72	2.82	0.242
Stage III	8.33	3.33	55.83	10.83	21.67	0	

### Relationship Between Microbial Profiles and Periodontitis Stages

To examine the relationship between *P. gingivalis* levels and periodontitis progression as well as the abundance of other bacteria, we sequentially analyzed the keystone and accessory pathogen levels in patients with different periodontitis stages and characteristics. As shown in **Table 6**, more samples from patients with periodontitis stage III exhibited higher levels of total bacteria (54.17% >10^9^) and *P. gingivalis* (44.17% >10^6^) than samples from stage II patients, the latter with 46.48% >10^9^ for total bacteria and 38.03% >10^6^ for *P. gingivalis*. However, these differences are not statistically significant. We also observed a similar trend for the *S. cristatus*/*P. gingivalis* ratios. Specifically, 46.48% of samples from stage II patients had ratios greater than 100, but only 35.83% of samples from stage III patients had ratios greater than 100. We observed significantly higher levels of *T. denticola* in samples from stage III patients than in those from stage II patients (66.28% vs. 43.90% >10^3^, *p* = 0.016). These results are consistent with our observation that samples with higher *T. denticola* levels also possessed higher levels of *P. gingivalis* (mean = 8.86 × 10^7^) than samples with lower *T. denticola* levels (mean = 5.10 × 10^6^) (*p* = 0.0078) (**Table 4**). These findings indicate that co-colonization of *P. gingivalis* and *T. denticola* may be associated with periodontitis progression. We also examined correlation between other independent variables, including body mass index, smoking, and diabetes, and severities of periodontitis. We did not found significant influence of these variables on stages of periodontitis in this cohort (**Table 6**).

**Table 6:** Correlation between oral bacterial levels or independent variables and periodontal disease stages.

Bacterial levels	Periodontitis Stages^a^ (%)		*P* -value
	II	III	
Total bacteria			
<10^9^	53.52	45.83	0.304
>10^9^	46.48	54.17	
*P. gingivalis*			
<10^6^	61.97	55.83	0.406
>10^6^	38.03	44.17	
*T. forsythia*			
<5x10^5^	43.66	46.67	0.687
>5x10^5^	56.34	53.33	
*T. denticola*			
<10^3^	56.10	33.73	0.0165
>10^3^	43.90	66.28	
*F. nucleatum*			
<2.5x10^6^	50.70	55.00	0.565
>2.5x10^6^	49.30	45.00	
Ratio of *S. cristatus*/*Pg*			
<100	53.52	64.17	0.146
>100	46.48	35.83	
Independent variables			
Body mass index			
<25	30.95	69.05	0.318
>25	39.44	60.56	
Smoking			
No	39.86	60.13	0.182
Yes	28.57	71.43	
Diabetes			
No	36.97	63.03	0.884
Yes	38.46	61.54	

a Periodontitis stages: Based on 2017 World Workshop classification.

In addition, we examined the correlations among these oral bacteria. Levels of *P. gingivalis*, *T forsythia*, *T. denticola*, and *F. nucleatum* in the samples positively correlated with the level of total bacteria to various extents (**Table 7**). The level of *P. gingivalis* correlated most strongly with the level of total bacteria (*r* = 0.66), followed by *T. forsythia* (*r* = 0.59), *F. nucleatum (r* = 0.29), and *T. denticola* (*r* = 0.18). Interestingly, *T. forsythia* exhibited a relatively high degree of correlation with *P. gingivalis*, *T. denticola*, and *F. nucleatum* (*r* > 0.45), suggesting the importance of *T. forsythia* in periodontal dysbiosis. In contrast, *P. gingivalis* showed weaker correlations with *T. denticola* (*r* = 0.18) and *F. nucleatum* (*r* = 0.15). We did not observe any significant correlation between *T. denticola* and *F. nucleatum*.

**Table 7:** Correlation between levels of bacterial species.

Bacterial species	Pearson Correlation Coefficients (*r*)/*p*-value				
	Total bacteria	*P. gingivalis*	*T. forsythia*	*T. denticola*	*F. nucleatum*
Total bacteria	1.00	0.66/<0.0001	0.59/0.0001	0.18/0.0001	0.29/0.0001
*P. gingivalis*	0.66/0.0001	1.00	0.54/0.0001	0.18/0.0458	0.15/0.0429
*T. forsythia*	0.59/0.0001	0.54/0.0001	1.00	0.47/0.0001	0.46/0.0001
*T. denticola*	0.18/0.0386	0.18/0.0458	0.47/0.0001	1.00	0.12/0.1636
*F. nucleatum*	0.29/0.0001	0.15/0.0429	0.46/0.0001	0.12/0.1636	1.00

## Discussion

Previous studies based on the 2009–2010 NHANES demonstrated periodontitis incidence to be significantly higher in AAs (58.6%) and HAs (59.7%) than in CAs (42.6%) (Thornton-Evans et al., 2013). Here, we examined the potential risk factors for periodontal health disparities by comparing the abundance of several well-studied bacteria in dental plaque samples from periodontitis patients of different racial/ethnic backgrounds. We detected a significantly higher bacterial mass in the AA patients than in the CA and HA patients. Additionally, *P. gingivalis* levels were much higher in the AA and HA patients than in the CA patients, rendering *P. gingivalis* levels a potential risk factor for periodontitis progression in the AA and HA populations. Our findings also indicate the involvement of *S. cristatus* in regulating *P. gingivalis* levels. We previously reported an antagonistic relationship between *S. cristatus* and *P. gingivalis* (Xie et al., 2000). We identified arginine deiminase (ArcA), a surface protein of *S. cristatus*, as the signaling molecule to which *P. gingivalis* responds by repressing *fimA* gene expression and protein production (Xie et al, 2007; Wu and Xie, 2010). Our previous study on 13 subjects with periodontitis revealed the possibility of an inverse relationship between the number of *S. cristatus* cells and that of *P. gingivalis* cells in dental plaques, suggesting that *S. cristatus* may benefit the host by antagonizing the colonization and accumulation of *P. gingivalis* (Wang et al., 2009). This study with larger sample size further revealed significantly lower ratios of *S. cristatus* to *P. gingivalis* in samples from the AA and HA patients than in those from the CA patients, thereby establishing the association between the *S. cristatus/P. gingivalis* ratio and disparity in periodontitis. Consistent with our previous discovery of significantly higher *arcA* expression in *S. cristatus* than in *Streptococcus gordonii* (Lin et al., 2008), we did not find a negative correlation between *S. gordonii* and *P. gingivalis* (data not shown). Taken together, our findings suggest that these two streptococcal species play distinct roles in the highly orchestrated dental plaque formation. Furthermore, results from these clinical studies provide a rationale for eliminating *P. gingivalis* from oral microbial communities using synthesized peptide analogs derived from *S. cristatus* ArcA.

In this study, *T. forsythia* appeared to be the most closely correlated to *P. gingivalis* in dental plaques. Levels of both *P. gingivalis* and *T. forsythia* increased when the *S. cristatus/P. gingivalis* ratio was low. These results agree with our previous observation that levels of *P. gingivalis* and *T. forsythia* were correlated in multispecies biofilms using an *ex-vivo* binding assay (Ho et al., 2018). Several clinical studies also reported a strong co-occurrence relationship among *P. gingivalis*, *T. forsythia*, *T. denticola*, and *F. nucleatum* in subgingival plaques (Gmur et al., 1989; Wara-aswapati et al., 2009; da Silva-Boghossian et al., 2011). Here, we confirmed the positive correlations among these bacterial species in all 191 dental plaque samples, though with varying degrees of coefficiency. Interestingly, *T. forsythia* showed higher correlations with *P. gingivalis*, *T. denticola*, and *F. nucleatum* compared to those among other bacterial species. A recent clinical study used immunohistochemistry and qPCR to show that *P. gingivalis* and *T. forsythia* aggregated in dental plaques and periodontal tissues from periodontitis patients (Rajakaruna et al., 2018). The interaction between *F. nucleatum* and *T. forsythia* is well established; it likely involves the hydrolyzation of β-glucans by *T. forsythia* β-glucanase (Sharma et al., 2005; Honma et al., 2018). However, direct interaction between *T. forsythia* and *T. denticola* has not been revealed. In an *in vitro* polymicrobial biofilm study using *P. gingivalis*, *T. denticola*, and *T. forsythia*, Zhu *et al.* found a significant increase in the total biovolume for *P. gingivalis* and *T. denticola* in polymicrobial microcolonies compared to single-species biofilms. Conversely, only a few *T forsythia* cells were detected in the polymicrobial biofilms (Zhu et al., 2013). A study by Hashimoto *et al.* revealed coaggregation of *P. gingivalis* and *T. denticola* mediated by a *P. gingivalis* fimbrial protein and *T. denticola* dentilisin (Hashimoto et al., 2003). Here, our results support a recently proposed concept that bacterial properties within oral microbial communities are context-dependent (Hajishengallis and Lamont, 2021). Particularly, we showed that *S. cristatus* may regulate the levels of *P. gingivalis* and *T. forsythia* in dental plaques, and that *T. denticola* was only detected in dental plaques with relatively high levels of *P. gingivalis*.

In conclusion, characteristics of periodontal dysbiosis including increase in levels of *P. gingivalis* and its association with *T. forsythia*, *T. denticola*, and *F. nucleatum* are potential risk factors of disparities in periodontal health and periodontitis severity. Moreover, *S. cristatus* controls *P. gingivalis* levels in oral microbiota and plays an important role in regulating the virulence potential of multispecies communities.

## Data Availability Statement

The original contributions presented in the study are included in the article/supplementary material. Further inquiries can be directed to the corresponding authors.

## Ethics Statement

The studies involving human participants were reviewed and approved by the Committee for the Protection of Human Subjects of the University of Texas Health Science Center at Houston. The patients/participants provided their written informed consent to participate in this study.

## Author Contributions

HX and B-YW conceived the study and supervised the project. TL and QC performed and verified the statistical analyses. B-YW, SS, H-WM, and LA enrolled study participants. M-HH and JH helped sample process. All authors discussed the results and contributed to the final manuscript.

## Funding

The study was supported in part by grant MD007586 from the National Institute on Minority Health and Health Disparities, United States of America. This work was supported by in part by Meharry RCMI MD007593 from NIMHD.

## Conflict of Interest

The authors declare that the research was conducted in the absence of any commercial or financial relationships that could be construed as a potential conflict of interest.

## Publisher’s Note

All claims expressed in this article are solely those of the authors and do not necessarily represent those of their affiliated organizations, or those of the publisher, the editors and the reviewers. Any product that may be evaluated in this article, or claim that may be made by its manufacturer, is not guaranteed or endorsed by the publisher.
